# Wound Gel Formulations Containing Poloxamer 407 and Polyhexanide Have *In Vitro* Antimicrobial and Antibiofilm Activity Against Wound-Associated Microbial Pathogens

**DOI:** 10.3390/microorganisms12112362

**Published:** 2024-11-19

**Authors:** Jeyachchandran Visvalingam, Nandadeva Yakandawala, Suresh Regmi, Adetola Adeniji, Parveen Sharma, Miloslav Sailer

**Affiliations:** Kane Biotech Inc., 290-100 Innovation Drive, Winnipeg, MB R3T 6G2, Canada

**Keywords:** chronic wounds, topical wound treatments, wound gel, antimicrobial activity, antibiofilm activity, poloxamer 407, pH, polyhexanide

## Abstract

Chronic wounds are often caused or exacerbated by microbial biofilms that are highly resistant to antimicrobial treatments and that prevent healing. This study compared the antimicrobial and antibiofilm activity of nine topical wound treatments, comprising gels with different concentrations of poloxamer 407 (20–26%) and different pH levels (4–6) and containing polyhexanide (PHMB) as an antimicrobial agent; the effects of pH on wound gels containing this agent have not been previously reported. The wound gel formulations were tested against six common wound-associated microbial pathogens: *Staphylococcus aureus*, *S. epidermidis*, *Pseudomonas aeruginosa*, *Escherichia coli*, *Acinetobacter baumannii*, and *Candida albicans*. Time-kill assays were used to assess antimicrobial activity against planktonic forms of each species, and a colony biofilm model was used to assess antibiofilm activity against existing biofilms as well as inhibition of new biofilm formation. Biofilm inhibition activity was also assessed in the presence of common wound dressing materials. Wound gels with higher pH levels exhibited stronger antimicrobial activity, while poloxamer 407 concentrations >20% negatively impacted antimicrobial activity. Wound gel formulations were identified that had antimicrobial, antibiofilm, and biofilm inhibition activity against all tested species *in vitro*. Biofilm inhibition activity was not affected by contact with common wound dressings. Further development of these wound gels may provide a valuable new option for the treatment and prevention of chronic wounds.

## 1. Introduction

Chronic wounds, also known as non-healing or complex wounds, are commonly defined as wounds that have not healed through the usual mechanisms within 3 months [1]. An estimated 1–2% of the population of developed countries will experience chronic wounds during their lifetime; this incidence is likely to rise due to the increasing prevalence of diabetes and other chronic diseases that affect wound healing [2,3,4]. In the US, chronic wounds affect 16.3% of Medicare beneficiaries, and the costs associated with acute and chronic wound treatment (including treatment of bacterial and other microbial infections that often impair wound healing) have been reported to range from $28.1 to $96.8 billion/year [2,5]. Cost-effective topical treatments with antimicrobial activity are thus needed to improve clinical outcomes and decrease the clinical and economic burden of chronic wounds.

While bacteria and other microbes can exist in free-floating planktonic or adherent biofilm form, biofilms are predominant in natural settings and are one of the major causes of wound chronicity [6]. Biofilms are communities of microbes attached to each other and to biotic or abiotic surfaces that are encased in an extracellular polymer matrix consisting of protein, polysaccharides, and/or extracellular DNA (eDNA), collectively called extra-polymeric substances (EPS). This EPS provides a protective coating against exogenous chemicals, allowing biofilm microbes to tolerate up to 1000-fold higher concentrations of antibiotic and antimicrobial agents compared to their planktonic counterparts, hindering attempts to treat chronic wounds with these agents [6,7]. In addition, biofilms enable microbes to evade the host immune system, causing persistent infections [8].

Biofilms within wounds can trigger a prolonged inflammatory phase, characterized by elevated levels of matrix metalloproteases and increased (neutral-to-alkaline) pH, that inhibits the normal wound-healing process. These changes in the pH of the wound affect many aspects of wound healing including protease activity, bacterial colonization and toxicity, angiogenesis, and oxygen release [9,10,11]. It is now accepted that creating a mildly acidic environment (pH 4–6) can mitigate these effects and facilitate wound healing, and wound care products that target pH modulation within this range have therefore been promoted as treatments for chronic wounds [12,13,14]. However, it has not yet been well established whether pH-modulating wound care products can impact biofilms specifically [15].

While pH is an important factor in wound chronicity, other aspects of the wound environment can also be modified to promote healing. For instance, maintaining a moist wound environment can accelerate healing of both acute and chronic wounds and aid in the development of new tissue by facilitating cellular growth and a rapid increase in collagen within a healthy noncellular matrix. Wound care products that can provide a moist wound environment—often containing water mixed with polymers such as poloxamers to increase viscosity and facilitate adherence of dressing materials to the wound surface—have been shown to improve wound healing, prevent biofilm formation, and promote autolytic debridement of wounds [16,17,18]. In addition, hydrogels have been shown to accelerate the healing of diabetic oral ulcers and can potentially be useful tools to treat other types of wounds associated with chronic diseases [19].

A previous study assessed the antibiofilm properties of a poloxamer-based gel [16]; however, this product did not contain any antimicrobial agents significant to wound care and its pH was not reported. Other wound care product formulations containing the antimicrobial agent polyhexanide (PHMB) have also been tested against wound pathogen biofilms, but again the effects of pH were not evaluated [20,21]. To the best of the authors’ knowledge, this is the first study to evaluate the impact of poloxamer 407 content, PHMB, and pH on the antimicrobial and antibiofilm properties of a wound gel formulation.

Therefore, the objectives of the current study were to develop novel poloxamer 407-based wound gels that combine pH and moisture control with antimicrobial and antibiofilm activity, and to evaluate their efficacy against the common wound-associated bacteria *Staphylococcus aureus*, *S. epidermidis*, *Pseudomonas aeruginosa*, *Escherichia coli*, and *Acinetobacter baumannii*, and common wound-associated fungus *Candida albicans* [22,23,24].

## 2. Materials and Methods

### 2.1. Preparation of Wound Gel Formulations

Proprietary wound gel formulations for the treatment of wounds [25] were prepared with metal ion chelators, gelling agent poloxamer 407 (Spectrum Chemicals, New Brunswick, NJ, USA), a humectant, glycerol (Spectrum Chemicals), and PHMB (Carbosynth LLC, San Diego, CA, USA). A total of nine gel formulations were prepared, containing combinations of three poloxamer 407 concentrations (P1–P3, range 20–26%, with P1 being lowest) and three pH levels (K1–K3, pH range 4–6, with K1 being lowest). Poloxamer 407 was dissolved in reverse osmosis water by stirring in an ice bath or using a jacketed vessel cooled to 10–15 °C [26]. The pH of each gel formulation was adjusted using 30% citric acid solution (Spectrum Chemicals). Formulations with low (P1), medium (P2), and high (P3) poloxamer concentrations yielded a solid gel at ≥20 °C, ≥16 °C, or ≥13 °C, respectively, and remained as a gel at 35–37 °C.

### 2.2. Microbial Culture Preparation

*S. aureus* (ATCC 6538), *P. aeruginosa* (ATCC 15422), and *E. coli* (ATCC 25404) were obtained from Cedarlane Labs (Burlington, ON, Canada); *S. epidermidis* 1457 was provided by Dr. T. Romeo (University of North Texas Health Sciences Center, Fort Worth, TX, USA); and *A. baumannii* 63270 and *C. albicans* (KBI 117) were provided by Dr. G. Zhanel (Department of Medical Microbiology and Infectious Diseases, University of Manitoba, Winnipeg, MB, Canada). Trypticase soy broth (Becton Dickinson; Fisher Scientific, Ottawa, ON, Canada) and trypticase soy agar (Becton Dickinson; Fisher Scientific) were used as liquid and solid media, respectively, for the cultivation of *S. aureus*, *S. epidermidis*, and *P. aeruginosa*; Lennox broth (Becton Dickinson; Fisher Scientific) and Lennox agar (Becton Dickinson; Fisher Scientific) were used for the cultivation of *E. coli* and *A. baumannii*; and potato dextrose broth (Oxoid; Fisher Scientific) and potato dextrose agar (Oxoid; Fisher Scientific) were used for the cultivation of *C. albicans*. All microbial cultures were stored at −80 °C as stock cultures in the appropriate broth containing 15% glycerol (Sigma Aldrich, Oakville, ON, Canada). Working cultures were maintained on an appropriate agar medium (above) at 4 °C with monthly transfer, for a maximum duration of 3 months. A single colony from a streaked plate was transferred to 5 mL of an appropriate broth medium and incubated overnight (16–18 h) at 37 °C. The absorbance value of each overnight culture was measured at 600 nm (A600). *E. coli* and *A. baumannii* cultures with A600 of >0.7 and other cultures with A600 of 0.9–1.2 were used for antimicrobial and antibiofilm activity testing.

### 2.3. Antimicrobial Activity Assessment: Planktonic Microbes

Overnight cultures of test organisms grown to the A600 values indicated above were serially diluted 10, 100, and 1000-fold in the appropriate broth medium to obtain ~10^7^, 10^6^, and 10^5^ colony-forming units (CFU)/mL, respectively. Each wound gel formulation was chilled for ≥30 min at 2–8 °C before testing to reduce viscosity to a liquid state. Four hundred microliters of each of the diluted cultures was then mixed with 400 mg of each wound gel formulation and incubated for 1 h, 3 h, and 24 h at 37 °C without shaking. After liquid incubation, a 100 μL sample was mixed with a 100 μL neutralizer solution for PHMB [27], and two spots of 10 μL each were placed onto appropriate agar medium plates and incubated for 24 h at 37 °C. The formation of colonies on these plates after 24 h of incubation indicated viable cell survival. Experiments were conducted in triplicate and repeated twice (*n* = 6). The detection limit of this spot plating approach was 2 log CFU/mL; lack of colony growth at 10^5^, 10^6^, and 10^7^ CFU/mL was indicative of ≥3, ≥4, and ≥5 log CFU/mL reductions in viable cell numbers, respectively.

### 2.4. Antibiofilm Activity Assessment: Preformed Biofilms

The colony biofilm assay described by Hammond et al. [28] was used with modifications. Briefly, overnight cultures of each bacterial species were diluted 100-fold in the appropriate broth. A sterile nitrocellulose membrane (Sigma Aldrich) was placed in each well of a 12-well plate containing 2 mL of the appropriate agar medium for each species, and 10 µL of diluted culture was inoculated onto each membrane. Microwell plates were incubated at 37 °C for 24 h to allow biofilm formation. Two sets of three biofilm-containing membranes were allocated for each gel formulation, in which 400 mg of gel was applied to each membrane and incubated for 24 h at 37 °C. Biofilms grown on membranes with no gel application were used as controls. For the first set of three membranes, each membrane was washed twice in 2 mL PBS to remove planktonic and loosely attached cells, placed into a 14 mL tube containing 2 mL neutralizer solution for PHMB and five 5 mm glass beads, and vortexed at maximum speed for 1 min to remove biofilm-embedded microbial cells. Each suspension was then serially diluted to up to 10^−7^ and each dilution was plated onto an appropriate agar plate. Plates were incubated at 37 °C for 24–48 h and colonies were counted. Viable cell counts (log CFU/membrane) were determined for each organism. For the second set of three membranes for each gel formulation, each membrane was washed twice in 2 mL PBS, placed onto an appropriate agar plate, and the same gel formulation was re-applied and incubated for 24 h at 37 °C before membrane processing and viable cell counting, as before. Each triplicate experiment was repeated twice (*n* = 6).

### 2.5. Antibiofilm Activity Assessment: Inhibition of Biofilm Formation

These experiments were performed as for the preformed biofilm experiments, above, except that inoculated membranes were not incubated to allow biofilm formation prior to gel application. Gel treatment was maintained for 24 h at 37 °C.

### 2.6. Assessment of Biofilm Inhibition in the Presence of Wound Dressings

To simulate clinical application, the biofilm inhibition efficacy of a selected wound gel formulation was tested in the presence of common non-antimicrobial wound dressings: cotton (Dermacea, Covidien LLC, Mansfield, MA, USA), silicone (Mepilex, Mölnlycke Health Care, Oakville, ON, Canada), collagen (Puracol, Medline Industries LP, Mississauga, ON, Canada), hydrocolloid/super absorbent gel (Super Absorbent Dressing, McKesson, Richmond, VA, USA), polyurethane foam (Optifoam Basic; Medline Industries LP, Mississauga, ON, Canada), and alginate (MedVance, MedWay Inc., Suwanee, GA, USA). Cultures were prepared and membranes were inoculated as above. A set of three membranes was allocated for each of the following treatments: wound gel alone, wound dressing alone, wound gel plus wound dressing, and untreated control. For treatments that included a wound gel, 400 mg of gel was applied to each inoculated membrane; for treatments that included a wound dressing, a 1.5 cm × 1.5 cm piece of dressing was placed either over the gel or, if no gel treatment was included, directly onto the inoculated membrane. Each dressing piece was lightly pressed down to have complete contact with the gel or inoculated membrane. Plates were incubated for 24 h at 37 °C. Each membrane was then washed and processed as before, serially diluted, and plated onto appropriate agar plates. After incubation at 37 °C for 24 h, viable cell numbers (log CFU/membrane) were determined as before. Experiments were performed in triplicate.

### 2.7. Data Analysis

A scoring system of 1–5 was used to compare the antimicrobial activity of the nine gel formulations against planktonic forms of each microbial species, based on reduction in viable cell numbers at each treatment time, with higher scores indicating greater antimicrobial activity (Table 1). The total score for each formulation was calculated by adding the scores obtained for each formulation against each of the six microbial species tested at each timepoint (maximum score for each species, 12; maximum total score, 72).

For assessment of each gel formulation’s antibiofilm activity, viable cell numbers were analyzed using one-way ANOVA using GraphPad Prism 10 (GraphPad Software, Boston, MA, USA) and expressed as the mean (standard error of the mean [SEM]). The post-hoc Tukey test was used to assess differences between means, and a *p*-value of ≤0.05 was considered statistically significant. To further compare the overall antibiofilm activity of the gel formulations, a scoring system of 0–5 was used based on the reduction in viable cell numbers of a given microbial species after the first and (separately scored) second 24 h gel application. Scores of 0 were assigned to any formulation yielding ≤0.5 log CFU reduction at the tested timepoint; formulations yielding 1–5 log CFU reduction were assigned scores of 1–5, respectively. Total scores for each formulation were calculated by adding the individual scores for each species tested at each timepoint (maximum score per species, 10; maximum total score, 60).

For the analysis of the effect of dressings on biofilm inhibition, viable cell numbers were analyzed using one-way ANOVA using GraphPad Prism 10 (GraphPad Software) and expressed as the mean (SEM). The post-hoc Tukey test was used to assess differences between means, and a *p*-value of ≤ 0.05 was considered statistically significant.

## 3. Results

### 3.1. Antimicrobial Activity Against Planktonic Microbes

The antimicrobial activity scores of the nine wound gel formulations against planktonic microbes ranged from 48 (high poloxamer 407 content, low pH) to 62 (low poloxamer content, medium pH and medium poloxamer content, high pH) out of a maximum possible score of 72 (Table 2). For each of the three poloxamer 407 concentrations tested, antimicrobial activity was lowest at the most acidic pH. Among the three sets of formulations with the same pH, antimicrobial activity was lowest for the formulations with the highest poloxamer 407 content.

All formulations inactivated >5 log CFU of test organisms within 24 h, and most achieved this level of viable cell reduction in ≤1 h or ≤3 h (Table 3). Notable exceptions were the inactivation of *S. aureus* and *S. epidermidis* by formulations with lower pH levels and/or higher poloxamer levels, which took >3 h (Table 3).

### 3.2. Antibiofilm Activity Against Preformed Biofilms

Based on the above results, three wound gel formulations (P1K3, P2K3, and P3K3) were selected for further assessment in antibiofilm activity experiments. P2K3 and P3K3 were selected because they showed the highest antimicrobial activity against planktonic microbes at medium and high poloxamer 407 concentrations, respectively. Among the low poloxamer 407 content gels, P1K3 scored slightly lower than P1K2 (61 versus 62) but was selected for further study as it has the same pH as P2K3 and P3K3, allowing an analysis of the effect of poloxamer 407 content on antibiofilm activity.

**Figure 1:**
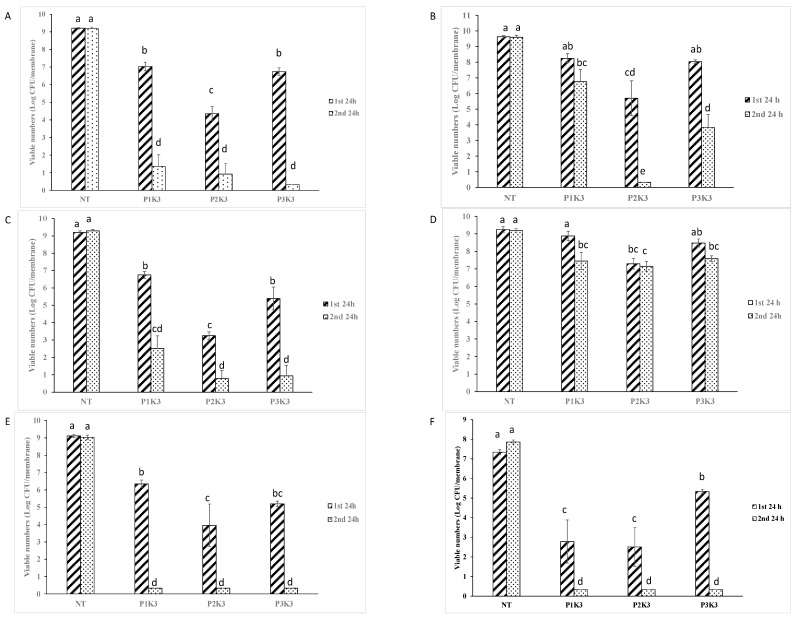
Antibiofilm activity of wound gel formulations against preformed biofilms of (**A**) *S. aureus*, (**B**) *S. epidermidis*, (**C**) *A. baumannii*, (**D**) *P. aeruginosa*, (**E**) *E. coli*, and (**F**) *C. albicans* after two successive 24 h application of the gel. NT, no treatment. Mean values identified by different alphabets are significantly different (*p* < 0.0001).

Species- and timepoint-specific data are shown in Figure 1. After the first 24 h gel application, formulation P2K3 caused a significantly greater reduction in the number of viable *S. aureus* biofilm cells (4.8 log CFU reduction) than did P1K3 or P3K3 (2–2.5 log CFU; *p* < 0.0001, 95% CI 1.353, 3.994 or CI −3.704, −1.063; Figure 1A). After the second 24 h gel application, all three gels caused a ≥8 log CFU reduction in viable cell numbers, with no significant differences between the treatments.

P2K3 also caused the largest first-application reduction in the viable numbers of other species: *S. epidermidis* (Figure 1B; NT vs. P2K3, 95% CI 2.216, 5.646; P2K3 vs. P1K3, or P3K3, 95% CI 0.4520, 4.653 or −4.436, −0.2348, *p* < 0.0001), *A. baumannii* (Figure 1C; NT vs. P2K3, 95% CI 4.391, 7.492, P2K3 vs. P1K3 or P3K3, 95% CI 1.711, 5.292 or −3.932, −0.3515, *p* < 0.0001), *P. aeruginosa* (Figure 1D; NT vs. P2K3, 95% CI 0.9474, 2.960, P2K3 vs. P1K3, *p* < 0.0001 95% CI 0.3500, 2.814; P2K3 vs. P3K3, *p* > 0.05), *E. coli* (Figure 1E; NT vs. P2K3, 95% CI 3.789, 6.531, P2K3 vs. P1K3, 95% CI 0.7166, 4.075 *p* < 0.0001; P2K3 vs. P3K3, *p* > 0.05), and *C. albicans* (Figure 1F; NT vs. P2K3, 95% CI 3.001–6.660, P2K3 vs. P3K3, 95% CI −4.940, −0.7152, *p* < 0.0001) compared to no treatment control, P1K3, or P3K3. The performance of the three formulations after the second application was generally more similar than after the first, but P2K3 was significantly more effective against *S. epidermidis* after the second application than were the other two formulations (*p* < 0.0001).

Formulation P2K3 had the highest overall antibiofilm activity score (53), while P1K3 had the lowest (38; Table 4).

### 3.3. Biofilm Inhibition Activity

**Figure 2:**
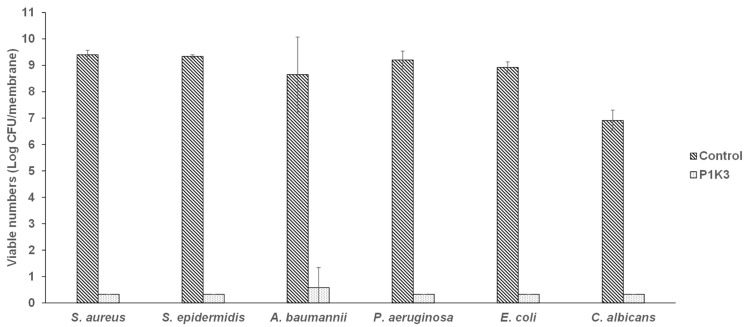
Biofilm inhibition activity of wound gel formulation P1K3 against six microbial species.

Assessing the activity of a highly effective wound gel formulation against preformed biofilms may not allow differentiation of the effects of the gel on different species or the effects of wound dressing materials (section below). Biofilm inhibition was therefore tested using P1K3, as this formulation had the lowest score when used against preformed biofilm. The numbers of viable cells harvested from untreated control biofilms of all tested bacterial organisms reached ≥9 log CFU after 24 h, while the numbers of *C. albicans* reached 7 log CFU (Figure 2). Application of P1K3 inhibited the growth of biofilms of all tested bacterial pathogens by ≥8 log CFU and of *C. albicans* by ≥6.5 log CFU.

### 3.4. Biofilm Inhibition Activity in the Presence of Wound Dressings

**Figure 3:**
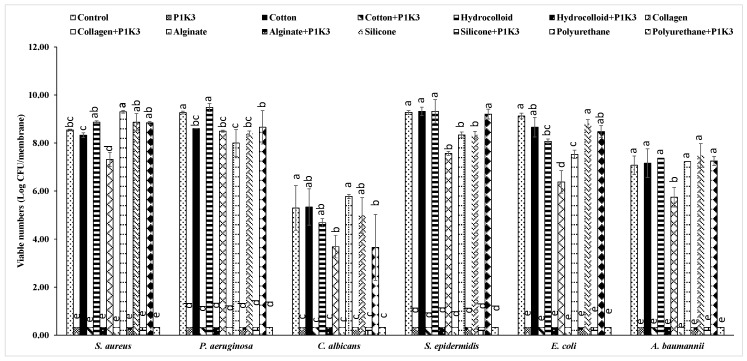
Biofilm inhibition activity of wound gel formulation P1K3 in the presence of common wound dressing materials. Mean values of each organism identified by different alphabets are significantly different (*p* < 0.0001).

Some of the wound dressing materials tested significantly reduced (*p* < 0.0006) the number of viable cells obtained from biofilms of certain microbial species by >1 log CFU compared to untreated control, even in the absence of P1K3 (Figure 3). Collagen dressings inhibited biofilm formation by all tested organisms compared to control by 1–3 log CFU (*p* < 0.0001); alginate dressings inhibited *P. aeruginosa*, *E. coli*, and *S. epidermidis* biofilm formation by 1–3 log CFU compared to control (*p* < 0.0001, 95% CI 0.2002, 1.347; 2.0565, 3.434; 1.434, 1.972, respectively); silicone dressings inhibited biofilm formation by *P. aeruginosa* and *S. epidermidis* by ~1 log CFU (*p* < 0.0004, 95% CI 0.2990, 1.446; 0.6640, 1.201, respectively); polyurethane dressings inhibited *C. albicans* biofilm formation by 1.6 log CFU (*p* < 0.0006, 95% CI 0.7383, 3.883); and hydrocolloidal dressings inhibited *E. coli* biofilm formation by 1 log CFU (*p* < 0.0004, 95% CI 0.3682, 1.747). However, adding P1K3 to any dressing material resulted in much stronger biofilm inhibition: the number of viable cells obtained from biofilms of each species in the presence of this formulation was reduced to below detectable levels, indicating that the biofilm inhibition activity of this gel was not affected by common wound dressing materials.

## 4. Discussion

Recent international consensus guidelines on the treatment of chronic wounds emphasize the use of topical antimicrobial treatments as one of the key elements for achieving better wound-healing outcomes [29,30]. In this study, nine different topical wound gel formulations were made and tested for antimicrobial and antibiofilm activity against six common wound-associated microbial pathogens. The nine hydrogel formulations combined three different concentrations of poloxamer 407 with three pH levels and included PHMB as an antimicrobial agent. As a pH of 4–6 has been shown to be ideal for wound healing, control of microbial colonization, and modulation of harmful proteases [12,13,14], all gel formulations were formulated within this range. The lowest antimicrobial activity scores against planktonic bacteria were observed for the gels with the most acidic pH at each polymer concentration. Higher antimicrobial effectiveness of PHMB with increasing pH has also been reported by others [31,32]; this effect was due to the polycationic nature of PHMB, where increasing pH can increase the charge density of negatively charged components of the microbial cell membrane and microbial enzymes, which in turn can increase the ability of PHMB to bind to and inactivate microbial cells [33]. The increasing proportion of negatively charged citrate molecules at higher pH levels may also render microbial cells more sensitive to PHMB [34]. A correlation was also observed between poloxamer 407 content and antimicrobial activity, with lower antimicrobial activity scores observed for gels with higher poloxamer 407 concentrations. This effect was likely attributable to the reduced diffusion of PHMB in more viscous gels with higher poloxamer 407 content, as gelling temperature decreased with increasing poloxamer 407 content, leading to prolonged gel dissolution time and diffusion of PHMB [35,36]. Controlling planktonic microbes in acute wounds may be necessary in order to prevent biofilm formation that can lead to wound chronicity [37]. Early application of an antimicrobial wound gel that can effectively and quickly reduce viable planktonic microbial numbers by >5 log CFU may therefore have clinical utility by preventing wound chronicity.

In cases where biofilms are already present in a wound, it is necessary to disrupt the protective EPS to enable antimicrobial agents to access and inactivate the microbial cells embedded therein [17]. Three high-pH gel formulations with different poloxamer concentrations that performed well against planktonic bacteria also exhibited activity against preformed biofilms. Overall, the formulation with the medium poloxamer 407 content was found to be the most effective at reducing the number of viable cells obtained from treated preformed biofilms. The formulation with the highest poloxamer 407 content had more antibiofilm activity than did the formulation with the lowest poloxamer 407 content, despite the latter exhibiting stronger antimicrobial activity against planktonic bacteria. These results suggest that poloxamer 407 content can impact wound gel effectiveness against preformed biofilms and indicate that wound gels should use a balanced poloxamer 407 concentration that is effective against planktonic bacteria but that also allows sufficient diffusion of PHMB to inactivate biofilms.

Wound gels need to not only reduce the bioburden of planktonic microbes and biofilms within wounds but also to provide ongoing inhibition of biofilm reformation by any surviving or newly colonizing cells. A wound gel formulation with high antimicrobial activity against planktonic bacteria but relatively low effectiveness against preformed biofilms (P1K3) was chosen for biofilm inhibition activity assays. This formulation effectively inhibited the formation of biofilms of all tested organisms, indicating that even a gel formulation with relatively low effectiveness against preformed biofilms can strongly inhibit new biofilm formation.

Cross-contamination of surgical wounds can lead to new microbial growth and biofilm formation, leading to delayed wound healing [17]. Under typical clinical conditions, wounds are covered with a dressing after topical antimicrobial application to prevent such cross-contamination [38]. Wound gels thus need to retain their antimicrobial and antibiofilm activity when in contact with common dressing materials. The gel formulation tested was able to inhibit the formation of biofilms of all tested organisms to below detectable limits in the presence of all major wound dressing material classes, suggesting that major wound dressings do not negatively affect the antimicrobial and antibiofilm properties of this prototype wound gel.

The current study used PHMB as the antimicrobial agent in all wound gel formulations. This agent was chosen for the study as it has been found to have a greater safety margin than other commonly used antimicrobials such as benzalkonium chloride, chlorhexidine, octenidine, and PVP-iodine [39,40]. As a cationic antimicrobial agent, PHMB can bind to negatively charged bacterial membrane components, leading to increased microbial cell permeability and loss of membrane integrity. Upon entry into the microbial cytoplasm, PHMB can disrupt bacterial metabolism and bind to DNA, ultimately causing microbial inactivation [40]. Because PHMB targets multiple cellular components, it is much less likely to cause antimicrobial resistance, as evidenced by the fact that PHMB has been in use for over 60 years with no evidence for the emergence of resistant microbial strains [41]. Considering that many antimicrobials currently used in wound care (such as silver, silver sulfadiazine, chlorhexidine, and benzalkonium chloride) have recently been identified as high- or medium-level antimicrobial resistance concerns by the US FDA and WHO [42], a PHMB-based wound gel with effective antimicrobial and antibiofilm properties would be highly valuable. Furthermore, since the gelling agent poloxamer 407 is a surfactant it can facilitate biofilm breakdown, exposing EPS-embedded microbes to the antimicrobial agent PHMB and leading and to their inactivation [43]. Evidence from this study also suggests that poloxamer 407 gel can facilitate the slow release of PHMB, thus maintaining its antimicrobial potential for longer periods [36].

In conclusion, this study provides evidence that the antimicrobial and antibiofilm activity of poloxamer-based antimicrobial wound gel formulations can be modulated by adjusting the poloxamer 407 content and pH. Furthermore, the formulations demonstrated antimicrobial and antibiofilm activity against major pathogens associated with chronic wounds, combat trauma, and burn injuries. The pH of all tested formulations fell within the optimal range of 4–6, which facilitates wound healing by modulating harmful protease activity and reducing microbial colonization or biofilm formation. All formulations were effective at reducing large bioburdens within 24 h, suggesting that the gels could be a valuable tool for reducing infections in acute wounds, such as surgical incisions. Further development of these prototype wound gels may provide new options for the treatment and prevention of chronic and acute wounds.

Future directions: This study evaluated the effect of poloxamer 407 content and pH on the antimicrobial and antibiofilm activity of different wound gel formulations. The assays used an *in vitro* colony biofilm assay that mimics the environmental conditions required for biofilm growth on a wound bed by allowing microorganisms to grow on a filter membrane supplied with nutrients diffused from below and in the presence of oxygen, but with little shear stress. This model has been used to evaluate wound care products previously [28,44]; however, it does not incorporate other biological components of wound environments such as blood/plasma, collagen, and skin tissue. Thus, further studies using *ex vivo* or *in vivo* models such as the porcine skin explant model or animal wound models, respectively, with or without different dressing types, would provide valuable additional information on the efficacy of wound gel prototypes in more clinically representative environments [17,45]. The use of microscopy to analyze the impact of wound gel formulations on biofilms [46] would further enhance understanding and may elucidate mechanisms of action of wound gel formulations. Furthermore, the selection of a final formulation will require additional biocompatibility studies, including cytotoxicity [45] and shelf-life studies, to evaluate safety and stability, an essential step in satisfying medical device regulators’ requirements. Therefore, future studies are planned in this direction.

## Figures and Tables

**Table 1:** Scoring system for antimicrobial activity assessment.

		Reduction in Viable Cell Numbers (log CFU)		
		≥3	≥4	≥5
Treatment time (hours)	1	3	4	5
	3	2	3	4
	24	1	2	3

**Table 2:** Antimicrobial activity against planktonic microbes.

Formulation	Poloxamer 407 Concentration ^A^	pH ^B^	Total Score ^C^
P1K1	Low	Low	52
P1K2	Low	Medium	62
P1K3	Low	High	61
P2K1	Medium	Low	55
P2K2	Medium	Medium	60
P2K3	Medium	High	62
P3K1	High	Low	48
P3K2	High	Medium	53
P3K3	High	High	58

^A^ Range: 20–26%. ^B^ Range: 4–6. ^C^ Combined score for all microbial species at all timepoints. Maximum score: 72.

**Table 3:** Antimicrobial activity of wound gel prototypes observed with different planktonic cell numbers.

Formulation	Time Taken to Cause Complete Inactivation of Different Numbers Planktonic Cells (h)																	
	*S. aureus* (Log CFU/mL)			*S. epidermidis* (Log CFU/mL)			*A. baumannii* (Log CFU/mL)			*P. aeruginosa* (Log CFU/mL)			*E. coli* (Log CFU/mL)			*C. albicans* (Log CFU/mL)		
	≥3	≥4	≥5	≥3	≥4	≥5	≥3	≥4	≥5	≥3	≥4	≥5	≥3	≥4	≥5	≥3	≥4	≥5
P1K1	24	24	24	3	24	24	3	3	3	3	3	3	3	3	3	1	1	3
P1K2	24	24	24	3	3	24	1	1	1	1	1	1	1	1	1	1	1	1
P1K3	3	3	24	3	3	24	1	1	1	1	1	1	1	1	1	3	3	3
P2K1	24	24	24	24	24	24	3	3	3	1	1	1	1	1	3	1	1	3
P2K2	24	24	24	24	24	24	1	1	1	1	1	1	1	1	1	1	1	1
P2K3	24	24	24	3	3	24	1	1	1	1	1	1	1	1	1	1	1	1
P3K1	24	24	24	24	24	24	3	24	24	1	1	1	3	3	3	1	3	3
P3K2	24	24	24	3	24	24	3	3	3	1	1	1	3	3	3	1	3	3
P3K3	24	24	24	3	24	24	1	1	1	1	1	1	3	3	3	1	1	1

**Table 4:** Activity of selected wound gel formulations against preformed biofilms.

Formulation	Total Score ^A^
P1K3	38
P2K3	53
P3K3	42

^A^ Combined score for all microbial biofilm species at all timepoints. Maximum score: 60.

## Data Availability

The original contributions presented in the study are included in the article, further inquiries can be directed to the corresponding author.
